# Aortic Pulse Wave Velocity Determined with Oscillometric Pulse Wave Analysis Algorithm Antares Is an Independent Predictor of Major Adverse Cardiovascular Events: A Prospective Cohort Study

**DOI:** 10.3390/jcm13237035

**Published:** 2024-11-21

**Authors:** Marcus Dörr, Harald Lapp, Stefan Richter, Alexander Stäuber, Martin Bahls, Stefan Gross, Marc-Alexander Ohlow, Siegfried Eckert, Franziska Stäuber, Matthias Wilhelm Hoppe, Johannes Baulmann

**Affiliations:** 1 Department of Internal Medicine B, University Medicine Greifswald, 17475 Greifswald, Germany; marcus.doerr@uni-greifswald.de (M.D.);; 2 German Center for Cardiovascular Research (DZHK), Partner Site Greifswald, 17475 Greifswald, Germany; 3 Department of Cardiology, Zentralklinik Bad Berka GmbH, 99437 Bad Berka, Germany; 4 Department of Cardiology, SRH Klinikum Burgenlandkreis GmbH, 06618 Naumburg, Germany; 5 Department of Medicine, Training and Health, Philipps University of Marburg, 35032 Marburg, Germany; 6 Department of Cardiology, SRH Wald-Klinikum GmbH, 07548 Gera, Germany; 7 Clinic for General and Interventional Cardiology/Angiology, Heart and Diabetes Center North Rhine-Westphalia, 32545 Bad Oeynhausen, Germany; 8 Department of Sports Medicine and Exercise Therapy, Chemnitz University of Technology, 09126 Chemnitz, Germany; 9 Department of Exercise Science, Philipps University of Marburg, 35032 Marburg, Germany; 10 Praxis Dres. Gille/Baulmann, 53359 Rheinbach, Germany

**Keywords:** pulse wave velocity, pulse wave analysis, Antares, oscillometry, MACE

## Abstract

**Background/Objectives**: Aortic pulse wave velocity (aPWV) is a well-established surrogate marker of arterial stiffness. The Antares algorithm offers a method for determining aPWV from oscillometric blood pressure waveforms without requiring additional inputs. This prospective study aimed to evaluate the association and prognostic value of aPWV, determined by Antares, in predicting major adverse cardiovascular events (MACE). **Methods**: In total, 240 patients (median age 69, 25.4% female) underwent oscillometric blood pressure measurements, from which aPWV was calculated using the Antares algorithm. MACE, comprising myocardial infarction, stroke, or all-cause mortality, occurred in 19.2% of patients during a median follow-up of 43 months. Survival analyses were performed using continuous aPWV values, a 10 m/s threshold, and aPWV quartiles. Kaplan–Meier curves and log-rank tests were used to compare survival across aPWV groups. Cox proportional hazards models were applied to assess the independent predictive value of aPWV. **Results**: Patients with aPWV < 10 m/s showed significantly higher event-free survival compared to those with aPWV ≥ 10 m/s (log-rank *p* = 0.044). Quartile analysis reinforced this, with the highest event rate in the highest aPWV quartile (log-rank *p* < 0.01). Multivariable analysis confirmed aPWV as an independent predictor of MACE (HR per 1 m/s: 1.24, 95% CI: 1.08–1.41; HR per 1 SD: 1.53, 95% CI: 1.17–2.00, *p* = 0.002). Adding aPWV to a risk model improved predictive accuracy (C-index 0.68 to 0.71). **Conclusions**: In the investigated cohort, aPWV derived using the Antares algorithm is an independent predictor of cardiovascular events. This non-invasive approach is promising for improving simple outpatient risk stratification and targeting preventive measures.

## 1. Introduction

Arterial stiffening, especially in the aorta, is a defining characteristic of arteriosclerosis [1]. The main clinical consequence of aortic stiffening is systolic hypertension, characterized by an increase in systolic and pulse pressure. These factors significantly contribute to the risk of stroke, coronary artery disease (CAD), and heart failure [1,2]. Pulse wave velocity (PWV) is the speed at which pressure waves move through the arteries and is a direct and widely accepted measure of arterial stiffness [1,3,4]. In addition to the fundamental factors of elastin and collagen, vascular smooth muscle cell tone and stiffness significantly influence arterial stiffness [1]. The mechanisms of arterial stiffening vary depending on the associated diseases, such as arterial hypertension, metabolic disorders, diabetes mellitus, chronic kidney disease (CKD), chronic low-grade inflammation, immune diseases, and cancer, although many determinants are common across these conditions [1]. Noteworthy, elevated arterial stiffness is increasingly recognized as an important marker for cardiovascular (CV) disease, and aortic PWV (aPWV) is a strong predictor of future CV events and all-cause mortality [1,5,6].

Although invasive methods for measuring arterial stiffness are restricted to a small number of high-risk patients undergoing catheterization, non-invasive techniques are more broadly applicable. These non-invasive methods for estimating aPWV fall into two categories: (i) Two-point measurement devices: These devices calculate the travel time difference of pulse waves between two separate sensor positions. Examples include the SphygmoCor (AtCor Medical Pty Ltd, Sydney, Australia), Complior Analyse (Alam Medical, Vincennes, France), and PulsePen (DiaTecne s.r.l., Milan, Italy). (ii) Single-point measurement devices: These devices assess the time difference between the forward and reflected pulse waves at a single location, using PWA algorithms. In this context, the Antares algorithm (Redwave Medical GmbH, Jena, Germany) is an example of a PWA algorithm used to estimate arterial stiffness indices, such as central blood pressure [7,8,9] and aPWV [10]. This algorithm can be incorporated into automated blood pressure (BP) monitors that use the oscillometric method for upper arm BP measurement. A key feature of Antares is its ability to enable oscillometric BP monitors to act as single-point measurement devices for aPWV assessment by utilizing only oscillometric pulse waves, without requiring additional input data like age or BP [10]. Although the algorithm has proven feasible for determining aPWV, its independent association with future CV events and its prognostic value have not been established yet. Thus, the objective of this prospective study was to evaluate the association and prognostic value of aPWV, as derived by the Antares algorithm, in a population of patients with CV disease.

## 2. Materials and Methods

### 2.1. Study Population

**Figure 1:**
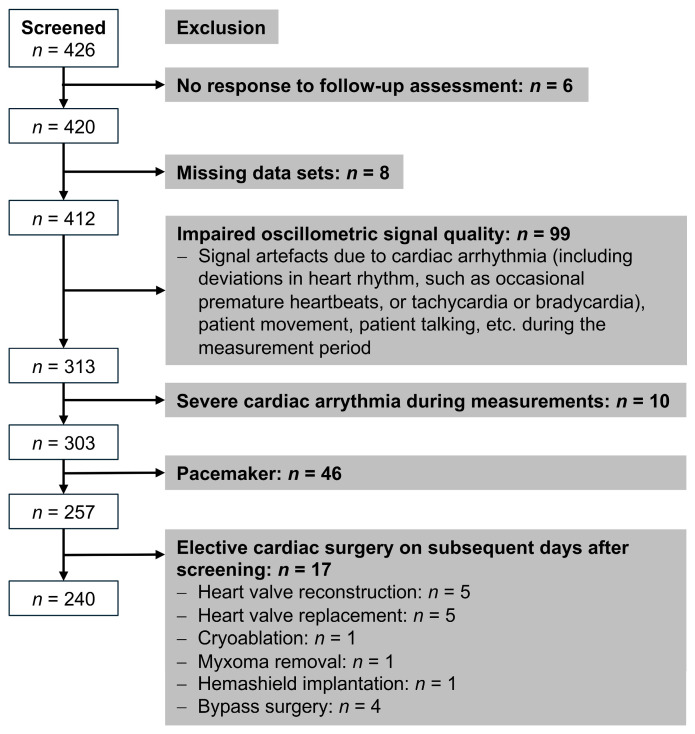
Flow chart for patient inclusion and exclusion.

This prospective study involved 426 patients undergoing elective cardiac catheterization at the Heart Center of Zentralklinik Bad Berka (Germany) from September 2018 to October 2020. Participants were required to be aged 18 years or older with a clinical indication for cardiac catheterization. Exclusion criteria for this analysis included no response to follow-up assessment, missing data sets, severe arrhythmia, unstable hemodynamic or clinical conditions, elective cardiac surgery within a few days (median 3 days, interquartile range 1–7.5 days) of the examination potentially impacting hemodynamics, impaired oscillometric signal quality, and the presence of a pacemaker. In total, 240 patients fulfilled the inclusion criteria (Figure 1). All patients continued to take their usual medication and provided written informed consent. Demographic and specific clinical patient characteristics were obtained from the medical records of the Heart Center of Zentralklinik Bad Berka.

### 2.2. Follow-Up Assessment and Study Endpoint

The follow-up assessment was conducted between January 2023 and June 2024. Patients were followed up by first contacting them with a written questionnaire by post. If there was no response, a telephone interview was carried out with the patient. If the patient was still unreachable, their general practitioner or, if available, the hospital record of subsequent hospitalizations was consulted. The composite endpoints of this study were three-point major adverse cardiovascular event (MACE) components: acute myocardial infarction (AMI), stroke, and all-cause death (ACD); these are the most utilized MACE components in observational research [11].

### 2.3. PWV Assessment

aPWV was calculated from non-invasively measured left upper-arm oscillometric pulse waveforms using the automated oscillometric BP monitor custo screen 400 (custo med GmbH, Ottobrunn, Germany) with the integrated oscillometric PWA algorithm Antares. All measurements were conducted in a supine position, with constant temperature and humidity, and without excessive ambient noise in the cardiac catheter laboratory. Patients were acclimated to the environment and free from any disturbing influences. Data acquisition took place during a period of undisturbed rest, free from acute hemodynamic interventions or recent medication changes, and in complete silence. No specific medication was administered immediately before the pulse wave recordings. The detailed examination procedure and the principle for obtaining pulse waves are both described and consistent with the invasive validation study for Antares central BP, as detailed elsewhere [7]. Oscillometric pulse waves were acquired during cuff deflation, with a deflation speed of 4 mmHg/s controlled by a regulated valve. This means that pulse waves generated during a standard oscillometric BP measurement can be used for PWA without modifying the standard BP pump operation. To avoid potential errors from peripheral BP measurements and surrogate input values, Antares relies solely on the raw signal of pulse waves during the deflation process provided by the oscillometric BP device, without incorporating any other data.

### 2.4. Statistics

All data were analyzed using SPSS version 22 (IBM Corp. Armonk, New York, NY, USA) and BlueSky Statistics version 10.3.2 (BlueSky Statistics LLC, Chicago, IL, USA). The continuous data were presented as median and interquartile range (IQR). Survival analysis included both continuous aPWV values and a categorical cutoff of 10 m/s, according to established guidelines [12,13]. Kaplan–Meier curves were generated to compare the two aPWV groups, with MACE probability distributions analyzed using the log-rank test. Additionally, comparisons were made by the 25th, 50th, and 75th percentiles of aPWV as cutoff-points. To assess the independent association of aPWV with the composite endpoint, a multivariable Cox regression analysis was conducted. Following the disjunctive cause criterion [14], the covariates age, sex, body mass index (BMI), smoking status, diabetes mellitus, CKD, CAD, heart failure, arterial hypertension, dyslipoproteinemia, and brachial systolic BP were initially included as set of potential confounders, as they are potentially common causes either of the exposure (aPWV), the outcome (AMI, stroke, all-cause mortality), or both. Backward elimination (LR method) was employed to systematically refine the model to retain only the minimal sufficient confounder set, thereby improving model parsimony. This approach helps to minimize the risk of uncontrolled confounding and enhances the reliability of causal inference. The proportional hazards assumption was tested and confirmed using Schoenfeld residuals. To enable comparisons with other studies and to assess the relative impact of aPWV, the aPWV values were standardized and included in Cox regression analysis. Standardization involved calculating z-scores for each aPWV measurement. This was carried out by subtracting the mean aPWV value from each individual aPWV measurement and then dividing the result by the standard deviation of aPWV values within the study population.

To assess the incremental prognostic value of aPWV, Harrell’s C-statistic (C-index) was utilized, as it is the most widely used metric for evaluating the performance of prognostic models in survival analysis [15]. The INVEST (International Verapamil-SR/Trandolapril Study) score served as the baseline for patient risk classification [16]. This score, specifically developed for patients with hypertension and chronic stable CAD, is well-suited to the characteristics of our study population. It incorporates several key variables, including age, BMI, heart rate, systolic BP, and histories of myocardial infarction, heart failure, stroke, smoking, diabetes mellitus, peripheral artery disease, and CKD, with a total possible score of 21 points [16]. The INVEST score was originally designed and validated to predict a composite endpoint consisting of all-cause mortality, AMI, and stroke, which aligns with the composite endpoint used in our study. A *p*-value < 0.05 was considered statistically significant for all analyses.

## 3. Results

### 3.1. Baseline Data

All 240 patients included in the study were Caucasian, with 25.4% being female. Among them, seven patients (2.9%) were under 50 years old, 120 patients (50.0%) were between 50 and 70 years old, and 113 patients (47.1%) were older than 70 years old. Selected baseline characteristics are detailed in Table 1. The median follow-up period was 43 months, during which 19.2% reached the combined MACE endpoint (*n* = 46 events in total; *n* = 35 with ACD, *n* = 4 with AMI, and *n* = 7 with strokes). The INVEST score classified 98 patients (41%) as low risk, 63 patients (26%) as intermediate risk, and 79 patients (33%) as high risk. Table S1 presents the characteristics of patients with and without MACE.

### 3.2. Survival Analysis

**Figure 2:**
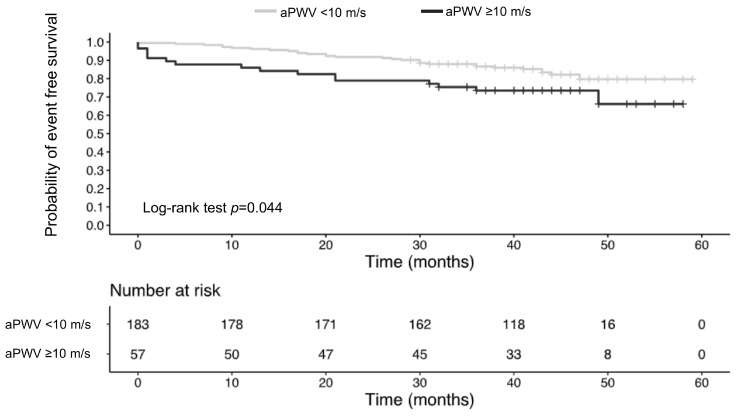
Kaplan–Meier plot of event-free survival for two groups, divided at the aPWV threshold of 10 m/s. aPWV, aortic pulse wave velocity.

**Figure 3:**
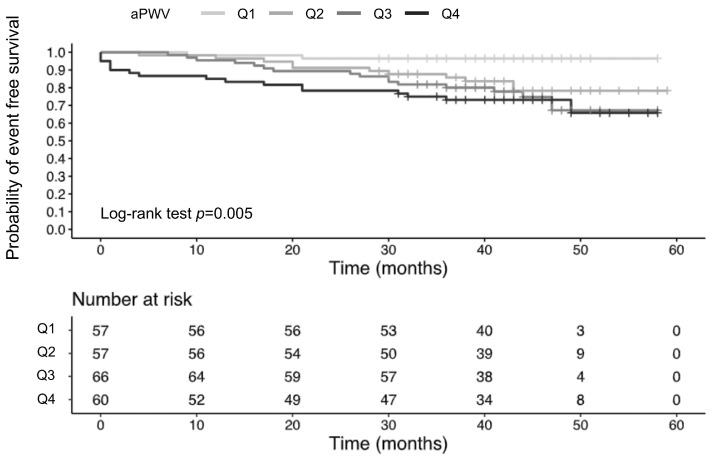
Kaplan–Meier plot illustrating event-free survival across four quartile groups based on Antares aPWV (m/s): Q1 (<7.5 m/s), Q2 (7.5- < 8.6 m/s), Q3 (8.6- < 9.9 m/s), and Q4 (≥9.9 m/s). Log-rank test between groups: Q1 vs. Q2, *p* = 0.01; Q1 vs. Q3, *p* = 0.002; Q1 vs. Q4, *p* < 0.001; Q2 vs. Q3, *p* = 0.465; Q2 vs. Q4, *p* = 0.198; Q3 vs. Q4, *p* = 0.560; aPWV, aortic pulse wave velocity.

As illustrated in Figure 2, patients with an aPWV below 10 m/s had a significantly higher event-free survival probability compared to those with an aPWV of 10 m/s or more (log-rank test, *p* = 0.044). As depicted in Figure 3, log-rank test identified statistically significant differences among quartile groups (*p* = 0.005). Patients with an aPWV above the 75th percentile (≥9.9 m/s) exhibited the highest incidence of MACE, reaching 28.3% (vs. Q1: 3.5%). The Log-rank test revealed significant differences between the first quartile (Q1) and the remaining quartiles (Q2–Q4), but no significant differences were observed among the groups Q2–Q4. Considering the INVEST score risk groups (low, intermediate, and high), the incidence of the combined outcome was 8%, 19%, and 33%, respectively (Figure S1; log-rank test, *p* < 0.001).

The multivariable Cox proportional hazards regression analysis identified a significant independent association of aPWV after adjustment for diabetes mellitus, CKD, and heart failure (Table 2). Each 1 m/s increase in aPWV was associated with an adjusted hazard ratio (HR) of 1.24 (95% confidence interval [CI]: 1.08–1.41; *p* = 0.002). Additionally, a 1 standard deviation (SD, 2.01 m/s) greater aPWV was related with an HR of 1.53 (95%CI: 1.17–1.99; *p* = 0.002).

Incorporating aPWV into the risk model with INVEST score improved both the predictive performance (C-index) and model fit (Akaike Information Criterion, AIC; Bayesian Information Criterion, BIC) moderately. The C-index increased from 0.68 to 0.71, while both AIC and BIC values decreased, indicating that aPWV enhances the prediction of CV risk, as shown in Table 3. Furthermore, in the combined Model 1, aPWV was independently associated with MACE, with an HR of 1.16 per 1 m/s increase (*p* < 0.001). In the analysis using standardized aPWV values, each 1 SD increase was associated with an HR of 1.35 (95% confidence interval: 1.04 to 1.76).

## 4. Discussion

This prospective study demonstrates that aPWV, as determined by the oscillometric PWA algorithm Antares, is independently associated with MACE (stroke, myocardial infarction, and all-cause mortality) and has a statistically significant prognostic value for predicting these outcomes in patients with CV disease.

Consistent with previous research [5,6], our study underscores the independent association between higher arterial stiffness, as reflected by elevated aPWV, and an increased risk of CV events. Several studies have demonstrated that aortic stiffness predicts all-cause and CV mortality, as well as fatal and nonfatal coronary events and stroke, in patients with essential arterial hypertension [17,18,19], type 2 diabetes mellitus [20], CKD [21], the elderly [22], and the general population [23,24]. An independent predictive value of aPWV has also been found in high-risk patients with suspected CAD undergoing invasive coronary angiography [25], a population that closely resembles the one in our study. In the study by Hametner et al. [25], both invasive aPWV measurements and non-invasive aPWV estimates derived from age, systolic BP, and PWA demonstrated significant predictive value. Our study also identified an independent association between aPWV and MACE risk, which supports previous findings. Using the oscillometric PWA algorithm Antares, which relies solely on oscillometric pulse wave data, we found that each 1 m/s increase in aPWV corresponded to a 24% higher risk of MACE in a multivariable Cox regression analysis. When standardized to a standard deviation (SD) of 2.01 m/s, a 1 SD increase in aPWV was linked to a 53% higher risk of MACE. Our findings align with those of Hametner et al. [25], who reported HRs of 1.28 to 1.37 for aPWV per increase of 1 SD in univariate analysis and HRs of 1.18 to 1.89 for the combined endpoint in multivariable models. Siasos et al. [26] also found a comparable independent significant association of non-invasively measured aPWV with MACE (HR per 1 m/s: 1.29, 95% CI: 1.07–1.57) in patients with stable CAD [26]. Therefore, there is growing evidence of an association between aPWV and MACE, justifying our study.

In addition to aPWV, our analysis identified diabetes mellitus, CKD, and heart failure as independent predictors of MACE (Table 2). While age and BP, known risk factors for CV events and aPWV [1], were not statistically significant in our prediction model, their clinical relevance suggests potential bias. However, upon adjusting for age and BP, the association between aPWV and MACE strengthened (HR per 1 m/s: 1.34, 95% CI: 1.09–1.64), highlighting its independent predictive value beyond traditional risk factors (Table S2). This underscores the unique contribution of aPWV to risk stratification. Moreover, Antares-based aPWV demonstrated superior predictive performance compared to standard values of an oscillometric BP measurement. Including BP in the final model instead of aPWV resulted in reduced model performance (C-index: 0.68, AIC: 459.9, BIC: 467.3), emphasizing the added value of Antares aPWV in risk assessment (Table S3).

Previous research has demonstrated the value of incorporating aPWV into CV risk prediction models [23,25,27,28,29]. For example, Vlachopoulos et al. [27] showed that adding estimated aPWV (derived from age and BP) to the Framingham Risk Score (FRS) significantly improved its predictive performance for all-cause mortality in the Systolic Blood Pressure Intervention Trial (SPRINT) population (*n* = 9361, mean age 67.9 ± 9.5 years, 3332 women). The C-index increased from 0.67 to 0.69, and the net reclassification index (NRI) also improved with the inclusion of aPWV [27]. However, despite the NRI’s potential, its clinical utility remains under debate [30]. 

In studies involving apparently healthy individuals, the addition of aPWV has consistently enhanced risk prediction models. In fact, Vishram-Nielsen et al. [29] observed improvements in both the C-index and NRI when adding formula-based aPWV (estimated from age and BP) to the FRS and Systematic Coronary Risk Evaluation (SCORE) models. Similarly, Greve et al. [28] reported better risk classification, when estimated aPWV was included in the SCORE model; however, no significant increase in C-index was found in the MONICA10 cohort. These findings were further supported by Mitchell et al. [23], who demonstrated a higher C-index in the Framingham Heart Study, when non-invasive, tonometrically measured aPWV was incorporated into a standard risk factor model [23]. When taken together, these outcomes show that aPWV is a valuable addition to traditional risk prediction models for CV events in apparently healthy individuals.

Our findings corroborate previous research demonstrating that integrating aPWV into risk assessment models can enhance predictive accuracy. The INVEST score, a validated tool for predicting CV disease events in patients with arterial hypertension and chronic stable CAD, incorporates a range of readily available non-laboratory clinical variables [16]. By adding the Antares-derived aPWV to the INVEST score, we observed an increase in the C-index (from 0.679 to 0.709) and a decrease in both the AIC and BIC. These results suggest that aPWV provides additional prognostic information beyond traditional risk factors included in the INVEST score, enabling more precise individual CVD risk assessment.

Given the established prognostic significance of the 10 m/s threshold for carotid-femoral PWV (cfPWV), as recommended by European guidelines [12,13,31] and supported by a meta-analysis [32], applying this threshold to aPWV in our analysis revealed that patients with an aPWV ≥ 10 m/s had significantly lower event-free survival compared to those with aPWV values below this threshold. Our findings align with those of Hametner et al. [25], demonstrating that an aPWV threshold of 10 m/s is a reliable predictor of adverse events. Our findings align with those of Hametner et al. [25] demonstrating that an aPWV threshold of 10 m/s serves as a reliable predictor of adverse events. Their study, with a longer follow-up period of 4.3 years compared to our 3.6 years, involved a similar cohort, but had a slightly younger median age of 63 years. However, our analysis of aPWV quartiles demonstrated that patients with an aPWV below 7.5 m/s (Q1) experienced significantly higher event-free survival compared to those in higher quartiles (Figure 3). The incidence of MACE was lowest in Q1 at 3.5%, with a progressive increase observed across quartiles: 19.3% in Q2, 24.2% in Q3, and 28.3% in Q4. It is important to note that these differences in MACE incidence may be partially influenced by age variations among the quartile groups. The median age in our study population was 66 years in Q1 and Q2, 71 years in Q3, and 75 years in Q4. While patients’ age may account for the differences in event-free survival between Q1 and Q3-Q4, it does not fully explain the difference between Q1 and Q2. Our results indicate that patients with an aPWV greater than 7.5 m/s might have a higher risk of MACE compared to those with lower aPWV values. This observation aligns with the findings of Mitchell et al. [23], who reported a progressive increase in CV event risk with higher quartiles of aPWV in a cohort of apparently healthy individuals (mean age: 63 years). Notably, they found that individuals with an aPWV below 7.7 m/s had the lowest probability of MACE during a median follow-up of 7.8 years.

In summary, our findings support the use of aPWV as a non-invasive valuable marker of CV risk and an effective tool for refining risk assessment, especially for intermediate- and high-risk patients with hypertension and CAD. By improving risk stratification, aPWV might help tailor interventions more precisely for those at greater risk. However, despite strong evidence linking aortic stiffening with adverse CV outcomes and the benefits of including aPWV in risk models, several challenges hinder its widespread use in routine clinical practice. A key issue is the variability in measuring aPWV, which can lead to inconsistent results and difficulties in comparing findings across studies [33]. To address this, standardizing measurement techniques and reporting practices is essential. 

Although the Antares algorithm extends the available methods for determining aPWV, it mitigates many of the methodological challenges associated with invasive methods and non-invasive two-point measurement devices [34]. This is achieved through its highly standardizable, user- and patient-friendly approach, which requires no special operator training and can be seamlessly integrated into conventional cuff-based oscillometric BP measurement. By integrating the assessment of aPWV into routine BP measurement, this method offers a practical and non-invasive approach to arterial stiffness monitoring. 

While our findings are promising, further research is needed to fully elucidate the clinical utility of aPWV in risk stratification. Our prospective study, conducted in a well-characterized patient population, has several strengths, but also a few limitations. The relatively short follow-up period and moderate sample size, which primarily comprised older white European males with established CV disease, restrict the generalizability of our findings to younger individuals, women, and other ethnic groups. Additionally, as an observational study, we cannot establish causality, and there is potential for confounding by associated risk factors or unknown variables. While the Antares algorithm for determining aPWV has shown promise in terms of feasibility and clinical utility, a comprehensive validation study is still needed to solidify its application. Furthermore, the current study exclusively used a custo screen 400 device for data acquisition. This device-specific approach may limit the generalizability of the Antares algorithm’s performance, as signal quality can vary across different oscillometric BP systems. Given Antares’ potential for integration into various oscillometric BP measurement systems, future evaluations should assess its performance across a wider range of devices. Despite these limitations, our results offer important insights into the relationship between Antares aPWV and the risk of MACE, underscoring the need for further research and potential clinical applications.

## 5. Conclusions

This prospective study is the first to demonstrate an independent association between aPWV, as measured by the oscillometric PWA algorithm Antares applied on data recorded with a custo screen 400 device and MACE including stroke, acute myocardial infarction, and all-cause mortality in patients with CVD. Our findings align with previous research linking increased arterial stiffness to elevated CV risk and support the inclusion of aPWV in risk assessment models to improve predictive accuracy. These results highlight the prognostic significance of aPWV and emphasize the need for further research to clarify its clinical utility.

## Figures and Tables

**Table 1:** Baseline characteristics of the included patients.

Variable	*n* = 240
Male sex	179 (74.6%)
Age, years	69.0 (61.0–78.0)
Weight, kg	82.5 (74.3–95.0)
Height, cm	172.0 (167.0–178.0)
BMI, kg/m^2^	28.7 (25.0–31.4)
Arterial hypertension	215 (89.6%)
Dyslipidemia	94 (39.2%)
Diabetes mellitus	90 (37.5%)
Chronic kidney disease	22 (9.2%)
Prior stroke	19 (7.9%)
Prior myocardial infarction	60 (25.0%)
Patients undergoing PCI	118 (49.2%)
Chronic heart failure	73 (30.4%)
Coronary artery disease	141 (58.8%)
Smoking	54 (22.5%)
INVEST score (max. 21 points)	5.0 (4.0–7.0)
INVEST low risk group (0–4 points)	98 (40.8%)
INVEST intermediate risk group (5–6 points)	63 (26.3%)
INVEST high risk group (>7 points)	79 (32.9%)
Betablockers	183 (76.3%)
Calcium channel blockers	97 (40.4%)
ACE inhibitor or angiotensin-receptor blockers	116 (48.3%)
Diuretics	65 (27.1%)
Statins	180 (75.0%)
Mineralocorticoid receptor antagonists	37 (15.4%)
Heart rate, bpm	65.0 (58.0–75.0)
cSBP, mmHg	134.0 (119.2–149.3)
cDBP, mmHg	73.5 (65.3–80.9)
cMAP, mmHg	95.1 (87.7–104.8)
cPP, mmHg	59.6 (47.1–71.9)
bSBP, mmHg	140.5 (128.3–154.8)
bDBP, mmHg	81.0 (75.0–89.0)
bMAP, mmHg	101.0 (92.3–111.0)
bPP, mmHg	58.0 (50.0–67.8)
aPWV, m/s	8.6 (7.5–9.9)

Numbers are median (25–75 percentile) or number of patients (percentage). ACE, angiotensin-converting enzyme; aPWV, aortic pulse wave velocity determined with algorithm Antares; b, brachial blood pressure determined with oscillometry; BMI, body mass index; c, central (aortic) blood pressure determined with algorithm Antares; SBP, systolic blood pressure; DBP, diastolic blood pressure; MAP, mean arterial pressure; PP, pulse pressure; PCI, percutaneous coronary intervention; INVEST (International Verapamil-SR/Trandolapril Study) score.

**Table 2:** Results of the multivariable Cox regression analysis to determine the associations with major adverse cardiovascular events (MACE).

Variable	Hazard Ratio (HR)	95% Confidence Interval (CI)	*p* Value
aPWV per 1 m/s	1.24	1.08–1.41	0.002
aPWV per 1 SD	1.53	1.17–1.99	0.002
Diabetes mellitus, yes/no	2.23	1.24–4.02	0.008
Heart failure, yes/no	2.38	1.29–4.36	0.005
CKD, yes/no	3.25	1.62–6.51	<0.001

Multivariable Cox regression analysis using backward elimination (LR method), including the following variables: age, sex, body mass index, aortic pulse wave velocity (aPWV), brachial systolic blood pressure, hypertension, dyslipidemia, diabetes mellitus, chronic kidney disease (CKD), smoking, heart failure, coronary artery disease.

**Table 3:** Results of Cox regression models for the prediction of the combined endpoint (all-cause mortality, myocardial infarction, stroke), predictive power, and model fit.

Model	Variable	HR (95% CI)	*p* Value	C-index	AIC	BIC
0	INVEST score	1.32 (1.18–1.48)	<0.001	0.679	461.98	463.81
1	INVEST score	1.31 (1.16–1.47)	<0.001	0.709	459.11	462.77
	aPWV per 1 m/s	1.16 (1.02–1.33)	0.024			
	aPWV per 1 SD	1.35 (1.04–1.76)	0.024			

AIC, Akaike information criterion; BIC, Bayesian information criterion; CI, confidence interval; C-index, concordance; HR, hazard ratio; INVEST (International Verapamil-SR/Trandolapril Study) score; aPWV, aortic pulse wave velocity estimated with algorithm Antares.

## Data Availability

The original contributions presented in the study are included in the article/Supplemental Materials; further inquiries can be directed to the corresponding author.

## Supplementary Materials

The following supporting information can be downloaded at: https://www.mdpi.com/article/10.3390/jcm13237035/s1, Figure S1: Kaplan–Meier plot showing event-free survival across risk groups, categorized by the International Verapamil-SR/Trandolapril Study (INVEST) risk score. The risk groups are defined as low risk (0–4 points), intermediate risk (5–6 points), and high risk (>7 points). Table S1: Characteristics of the included patients with and without major adverse cardiovascular event (MACE). Table S2: Multivariable Cox Regression Model results for associations with major adverse cardiovascular events (MACE). Table S3: Results of Cox regression models for the prediction of the combined endpoint (all-cause mortality, myocardial infarction, stroke), predictive power and model fit.
