# Supplementary material for: Aortic Pulse Wave Velocity Determined with Oscillometric Pulse Wave Analysis Algorithm Antares Is an Independent Predictor of Major Adverse Cardiovascular Events: A Prospective Cohort Study
Source: J Clin Med. 2024 Nov 21;13(23):7035. doi: 10.3390/jcm13237035 (PMC11642281; doi:10.3390/jcm13237035)
Supplement: Supplementary file 1 [file jcm-13-07035-s001.zip › Supplementary Materials.pdf]

Journal of
Clinical Medicine

MDPI

Supplementary File

Aortic Pulse Wave Velocity Determined with Oscillometric Pulse Wave Analysis Algorithm Antares is an Independent Predictor of Major Adverse Cardiovascular Events: A Prospective Cohort Study

Marcus Dörr [1,2], Harald Lapp [3], Stefan Richter [4], Alexander Stäuber [1,5], Martin Bahls [1,2], Stefan Gross [1,2], Marc-Alexander Ohlow [6], Siegfried Eckert [7], Franziska Stäuber [8], Matthias Wilhelm Hoppe [9] and Johannes Baulmann [10]

[1] Department of Internal Medicine B, University Medicine Greifswald, 17475 Greifswald, Germany
[2] German Center for Cardiovascular Research (DZHK), Partner Site Greifswald, 17475 Greifswald, Germany
[3] Department of Cardiology, Zentralklinik Bad Berka GmbH, 99437 Bad Berka, Germany
[4] Department of Cardiology, SRH Klinikum Burgenlandkreis GmbH, 06618 Naumburg, Germany
[5] Department of Medicine, Training and Health, Philipps University of Marburg, 35032 Marburg, Germany
[6] Department of Cardiology, SRH Wald-Klinikum GmbH, 07548 Gera, Germany
[7] Clinic for General and Interventional Cardiology/Angiology, Heart and Diabetes Center North Rhine-Westphalia, 32545 Bad Oeynhausen, Germany
[8] Department of Sports Medicine and Exercise Therapy, Chemnitz University of Technology, 09126 Chemnitz, Germany
[9] Department of Exercise Science, Philipps University of Marburg, 35032 Marburg, Germany;
[10] Praxis Dres. Gille/Baulmann, 53359 Rheinbach, Germany; jbaulmann@yahoo.com

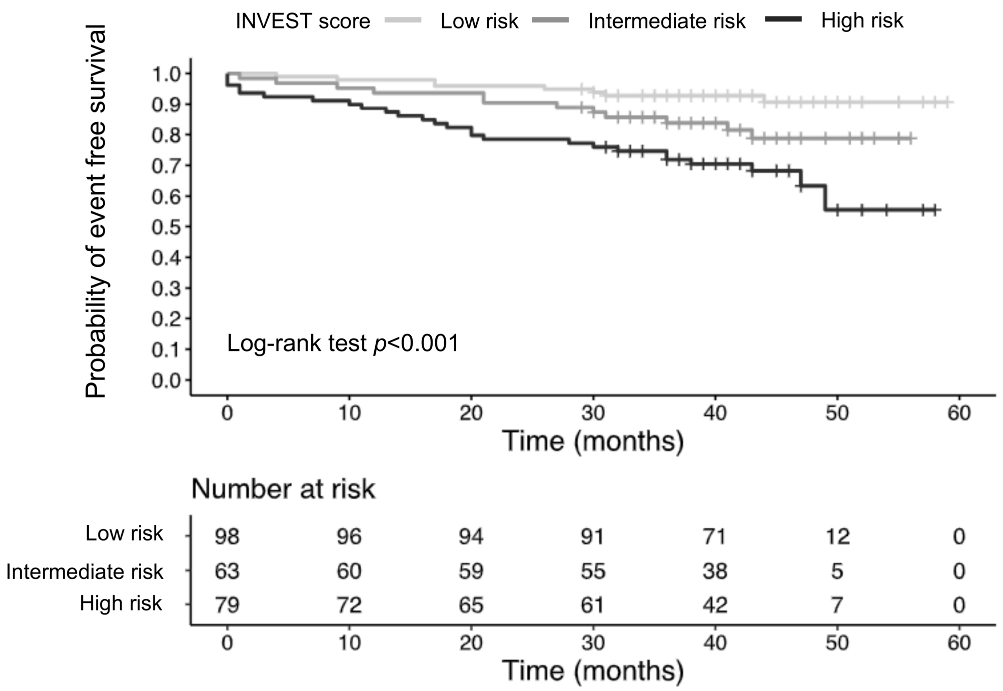

Figure S1. Kaplan–Meier plot showing event-free survival across risk groups, categorized by the International Verapamil-SR/Trandolapril Study (INVEST) risk score. The risk groups are defined as low risk (0-4 points), intermediate risk (5-6 points), and high risk (>7 points).

Table S1. Characteristics of the included patients with and without major adverse cardiovascular event (MACE).

Variable	MACE group	No MACE group	*p* value
n	46	194	0.438
Male sex	36 (78.3%)	143 (73.7%)	0.524
Age, years	75.5 (64.3-80.0)	68.0 (60.0-77.0)	**0.007**
Weight, kg	82.5 (70.0-94.8)	82.5 (75.0-95.0)	0.579
Height, cm	172.0 (168.0-176.8)	173.0 (167.0-179.0)	0.438
BMI, kg/m^2	29.4 (24.0-31.6)	28.7 (25.3-31.3)	0.751
Arterial hypertension	43 (93.5%)	172 (88.7%)	0.336
Dyslipidemia	24 (52.2%)	70 (36.1%)	**0.044**
Diabetes mellitus	26 (56.5%)	64 (33.0%)	**0.003**
Chronic kidney disease	12 (26.1%)	10 (5.2%)	**<0.001**
Prior stroke	4 (8.7%)	15 (7.7%)	0.828
Prior myocardial infarction	11 (23.9%)	49 (25.3%)	0.850
Patients undergoing PCI	25 (54.3%)	93 (47.9%)	0.453
Chronic heart failure	24 (52.2%)	49 (25.3%)	**<0.001**
Coronary artery disease	30 (65.2%)	111 (57.2%)	0.322
Smoking	13 (28.3%)	41 (21.1%)	0.298
INVEST score (max. 21 points)	7.0 (5.0-9.0)	5.0 (3.0-7.0)	**<0.001**
INVEST low risk group (0-4 points)	8 (17.4%)	90 (46.4%)	**<0.001**
INVEST intermediate risk group (5-6 points)	12 (26.1%)	51 (26.3%)	0.978
INVEST high risk group (>7 points)	26 (56.5%)	53 (27.3%)	**<0.001**
Betablockers	38 (82.6%)	145 (74.7%)	0.260
Calcium channel blockers	19 (41.3%)	78 (40.2%)	0.891
ACE inhibitor or angiotensin-receptor blockers	24 (52.2%)	92 (47.4%)	0.562
Diuretics	20 (43.5%)	45 (23.2%)	**0.005**
Statins	32 (69.6%)	148 (76.3%)	0.344
Mineralocorticoid receptor antagonists	8 (17.4%)	29 (14.9%)	0.680
Heart rate, bpm	68.5 (57.3-77.0)	65.0 (58.0-73.0)	0.374
cSBP, mmHg	138.6 (125.9-150.6)	133.3 (118.4-148.1)	0.140
cDBP, mmHg	71.3 (64.8-78.1)	73.8 (65.9-81.2)	0.564
cMAP, mmHg	95.2 (89.6-101.8)	95.1 (86.5-104.9)	0.809
cPP, mmHg	64.8 (52.5-75.4)	57.7 (46.2-71.2)	**0.031**
bSBP, mmHg	142.0 (131.3-155.5)	140.0 (128.0-154.0)	0.427
bDBP, mmHg	81.0 (75.0-86.8)	81.0 (75.0-89.0)	0.959
bMAP, mmHg	102.0 (95.0-111.8)	101.0 (92.0-110.0)	0.502
bPP, mmHg	61.0 (53.3-68.0)	57.5 (48.3-67.0)	0.311
aPWV, m/s	9.2 (8.4-10.7)	8.5 (7.2-9.8)	**0.002**

Numbers are median (25–75 percentile) or number of patients (percentage). ACE, angiotensin-converting enzyme; aPWV, aortic pulse wave velocity determined with algorithm Antares; b, brachial blood pressure determined with oscillometry; BMI, body mass index; c, central (aortic) blood pressure determined with algorithm Antares; SBP, systolic blood pressure; DBP, diastolic blood pressure; MAP, mean arterial pressure; PP, pulse pressure; PCI, percutaneous coronary intervention; INVEST (International Verapamil-SR/Trandolapril Study) score. Mann-Whitney U test was used for the analysis of numerical variables and chi-square test for categorical variables.

Table S2. Multivariable Cox Regression Model results for associations with major adverse cardiovascular events (MACE)

Variable	Model 1		Model 2	
	**HR (95% CI)**	***p* value**	**HR (95% CI)**	***p* value**
aPWV per 1 m/s	1.35 (1.08-1.69)	0.008	1.34 (1.09-1.64)	0.006
Diabetes mellitus, yes/no	2.15 (1.14-4.05)	0.018	2.34 (1.29-4.26)	0.005
Heart failure, yes/no	2.12 (1.13-3.98)	0.019	2.25 (1.22-4.15)	0.009
CKD, yes/no	2.94 (1.39-6.21)	0.005	2.92 (1.41-6.01)	0.004
Age per year	1.02 (0.99-1.06)	0.193	1.01 (0.98-1.04)	0.619
bSBP per 1 mmHg	0.99 (0.97-1.01)	0.265	0.99 (0.97-1.01)	0.202
Sex, male/female	1.23 (0.57-2.62)	0.599		
Hypertension	0.59 (0.16-2.24)	0.442		
CAD, yes/no	0.94 (0.48-1.86)	0.868		
Dyslipidemia, yes/no	1.47 (0.77-2.77)	0.240		
Smoking, yes/no	1.85 (0.88-3.91)	0.107		
BMI per 1 kg/m^2	0.99 (0.92-1.07)	0.826		

Multivariable Cox regression analysis (ENTER method) including the following variables:
Model 1 (full set of covariates): age, aortic pulse wave velocity (aPWV), body mass index (BMI), brachial systolic blood pressure (bSBP), coronary artery disease (CAD), chronic kidney disease (CKD), diabetes mellitus, dyslipidemia, heart failure, hypertension, sex, smoking.
Model 2: age, aortic pulse wave velocity (aPWV), brachial systolic blood pressure (bSBP), chronic kidney disease (CKD), diabetes mellitus, heart failure.

Table S3. Results of Cox regression models for the prediction of the combined endpoint (all-cause mortality, myocardial infarction, stroke), predictive power and model fit.

Model	Variable	HR (95% CI)	*p* value	C-index	AIC	BIC
0	aPWV per 1 m/s	1.24 (1.08-1.41)	0.002	0.723	452.25	459.56
	Diabetes mellitus, yes/no	2.23 (1.24-4.02)	0.008			
	Heart failure, yes/no	2.38 (1.29-4.36)	0.005			
	CKD, yes/no	3.25 (1.62-6.51)	<0.001			
1	bSBP per 1 mmHg	1.01 (0.99-1.02)	0.280	0.682	459.96	467.27
	Diabetes mellitus, yes/no	2.19 (1.22-3.94)	0.009			
	Heart failure, yes/no	2.32 (1.27-4.26)	0.007			
	CKD, yes/no	3.26 (1.64-6.51)	0.001			

AIC, Akaike information criterion; BIC, Bayesian information criterion; CI, confidence interval; C-index, concordance; HR, hazard ratio; INVEST (International Verapamil-SR/Trandolapril Study) score; aPWV, aortic pulse wave velocity estimated with algorithm Antares; bSBP, brachial systolic blood pressure.
